# Deciphering the molecular basis for photosynthetic parameters in Bambara groundnut (*Vigna subterranea* L. Verdc) under drought stress

**DOI:** 10.1186/s12870-023-04293-w

**Published:** 2023-05-30

**Authors:** Xiuqing Gao, Hui Hui Chai, Wai Kuan Ho, Sean Mayes, Festo Massawe

**Affiliations:** 1 School of Chemistry and Chemical Engineering, North University of China, Taiyuan, 030051 China; 2 Future Food Beacon, School of Biosciences, University of Nottingham Malaysia, Selangor Darul Ehsan, Jalan Broga, 43500 Semenyih, Malaysia; 3 Plant and Crop Sciences, School of Biosciences, University of Nottingham, Sutton Bonington Campus, Leics, Loughborough, LE12 5RD UK; 4 Crops for the Future (UK) CIC, 76-80 Baddow Road, Chelmsford, Essex, CM2 7PJ UK

**Keywords:** Bambara groundnut, Drought stress, QTL, Stomatal conductance, *F*_*V*_/*F*_*M*_

## Abstract

**Background:**

Assessment of segregating populations for their ability to withstand drought stress conditions is one of the best approaches to develop breeding lines and drought tolerant varieties. Bambara groundnut (*Vigna subterranea* L. Verdc.) is a leguminous crop, capable of growing in low-input agricultural systems in semi-arid areas. An F_4_ bi-parental segregating population obtained from S19-3 × DodR was developed to evaluate the effect of drought stress on photosynthetic parameters and identify QTLs associated with these traits under drought-stressed and well-watered conditions in a rainout shelter.

**Results:**

Stomatal conductance (*gs*), photosynthesis rate (*A*), transpiration rate (*E*) and intracellular CO_2_ (*Ci*) were significantly reduced (*p* < 0.05) while water use efficiency (WUE) was significantly increased (*p* < 0.05) under drought-stressed conditions. A strong linear correlation was observed between *gs,* WUE, *A, E* and *Ci* under both water regimes. The variability between different water treatment, among individual lines and the interaction between lines and environment for photosynthetic parameters provides resources for superior lines selection and drought resistant variety improvement. Significant QTL for *gs* and *F*_*V*_/*F*_*M*_ under well-watered conditions were mapped on LG5 and LG3, respectively, with more than 20% of the PVE, which could be considered as the major QTL to control these traits. Five clustered QTLs for photosynthetic traits under drought-stressed and well-watered conditions were mapped on LG5, LG6A, LG10 and LG11, respectively.

**Conclusions:**

Significant and putative QTLs associated with photosynthetic parameters and the effect of drought stress on these traits have been revealed by QTL linkage mapping and field experiment in the F_4_ segregating population derived from S19-3 × DodR in bambara groundnut. The study provides fundamental knowledge of how photosynthetic traits response to drought stress and how genetic features control these traits under drought-stressed and well-watered conditions in bambara groundnut.

**Supplementary Information:**

The online version contains supplementary material available at 10.1186/s12870-023-04293-w.

## Background

Drought is one of the major abiotic stresses, negatively impacting plant growth and reduce crop production worldwide [1, 2]. Drought stress caused significant changes in photosynthesis, *relative water content,* root and *shoot dry weight*, which are good indicators of drought monitoring in chickpea (*Cicer arietinum* L.) [3–5]. Three common bean (*Phaseolus vulgaris* L.) elite lines (NCB 226, SER 78, SER 125) showed superior levels of adaptation to drought stress conditions by remobilizing photosynthate to increase grain yield [6].

Bambara groundnut is an underutilised and drought-resistant leguminous crop with high protein content (16%-25%), which are mainly grown by subsistence farmers and served as an edible protein source in Africa [7–11]. It was shown that S19-3 landrace from Namibia experienced reduced respiration and stomata closure at a comparatively lower water threshold coupled with fast phenological development, short life cycle and early maturing proved to be among the mechanisms to ameliorate drought conditions [12, 13]. Three landraces of bambara groundnut collected from South Africa i.e., Brown, Red and Light Brown were reported to have reduced stomatal conductance of 1% – 8%, reduced *chlorophyll content index* (CCI), *plant height, leaf number*, reduced *leaf area index* and biomass accumulation of 5% – 8% and yield loss of 50% under water defict conditions [14]. Landrace Brown and Red showed higher *emergence rate, gs*, CCI and yielded more than Light Brown in response to water deficit conditions [14].

Similar to most of the underutilised and neglected crop species which have limited established breeding programmes due to lacking of commercial interest in breeding this crop and genetic improvement activities, landraces (mixture of genotypes) have remained as the main source of planting in bambara groundnut [12, 15–18]. Single plant descent (SPD) and single seed descent (SSD) has been highlighted to develop pure lines/genotypes of bambara groundnut [12]. Variation among genotypes with different drought response ability provides resources for breeders to select drought resistance varieties with high yield in bambara groundnut [13, 14, 19]. Strong genotypic variation was observed for many traits, i.e., *100-seed weight, harvest index, stomatal density* and *leaf area* in the F_5_ segregating population derived from Tiga Nicuru × DipC, facilitating the identification of superior and drought tolerant lines for advancement [20]. Kendabie et al. [21] reported that five segregating population, i.e., Ankpa4 × IITA-686 (reciprocal), Ankpa4 × DodR, Ankpa4 × DipC, S19-3 × Ankpa4 and IITA-686 × LunT were developed to create genetic linkage map and enhance trait dissection in bambara groundnut to accelerate crop breeding process. A genetic linkage map covering 1,040.92 cM across 11 linkage groups was constructed using 234 DArTseq-based SNP markers in the F_2_ segregating population from S19-3 × DodR [22]. Significant QTLs associated with *number of seeds per plant, number of double-seeded pod per plant, seed weight per plant* and *pod weight per plant* were mapped on LG4 with overlapping confidence intervals under well-watered conditions in the F_4_ population, which could be considered as major QTL involved in the control of these traits [22]. QTLs associated with stomatal density, length and conductance were co-located on chromosome II under greenhouse conditions in faba bean (*Vicia faba* L.) [23]. Lopez et al. [24] reported different* E* values but similar *A* values at the same QTL in Sorghum (*Sorghum bicolor* L. Moench). However, few studies have been reported for genetic analysis and variety development in the structured populations of bambara groundnut.

The first genome sequence of bambara groundnut has been assembled with 513 Mb in size and predicted 31,707 protein-coding genes [25]. However, the current bambara groundnut genome information and QTL mapping does not afford adequate resolution to identify genes. High density genetic linkage maps and QTL detection are very useful tools to identify genomic regions that may be responsible for target traits for MAS breeding of bambara groundnut [25]. In the present study, we evaluated the effect of drought stress on photosynthetic parameters and mapped QTLs for these traits under drought-stressed and well-watered conditions in the F_4_ segregating population derived from S19-3 × DodR in bambara groundnut. The study provided critical insights into how genetic features control photosynthetic traits in bambara groundnut under drought-stressed and well-watered conditions, which is also essential for crop improvement of bambara groundnut in response to drought stress.

## Results

### Photosynthesis response to drought stress during plant growth

The average of total reduction of soil moisture content under drought-stressed conditionswas 36.15% from 47 to 74 DAS. On average, soil moisture content declined by 0.41% per day at depth 300 mm and 0.31% per day at depth 400 mm over 28 days of drought (Supplementary Fig. S1). Significant reduction (*p* < 0.01) in soil moisture by 5.66%, 7.04% and 11.51% was observed under drought-stressed conditions compared to well-watered conditions at depth 100 mm, 200 mm and 300 mm, respectively. However, there was no significant difference (*p* > 0.01) for soil moisture content at depth 400 mm, 600 mm and 1000 mm between drought-stressed and well-watered conditions (Supplementary Fig. S2).

**Fig. 1:**
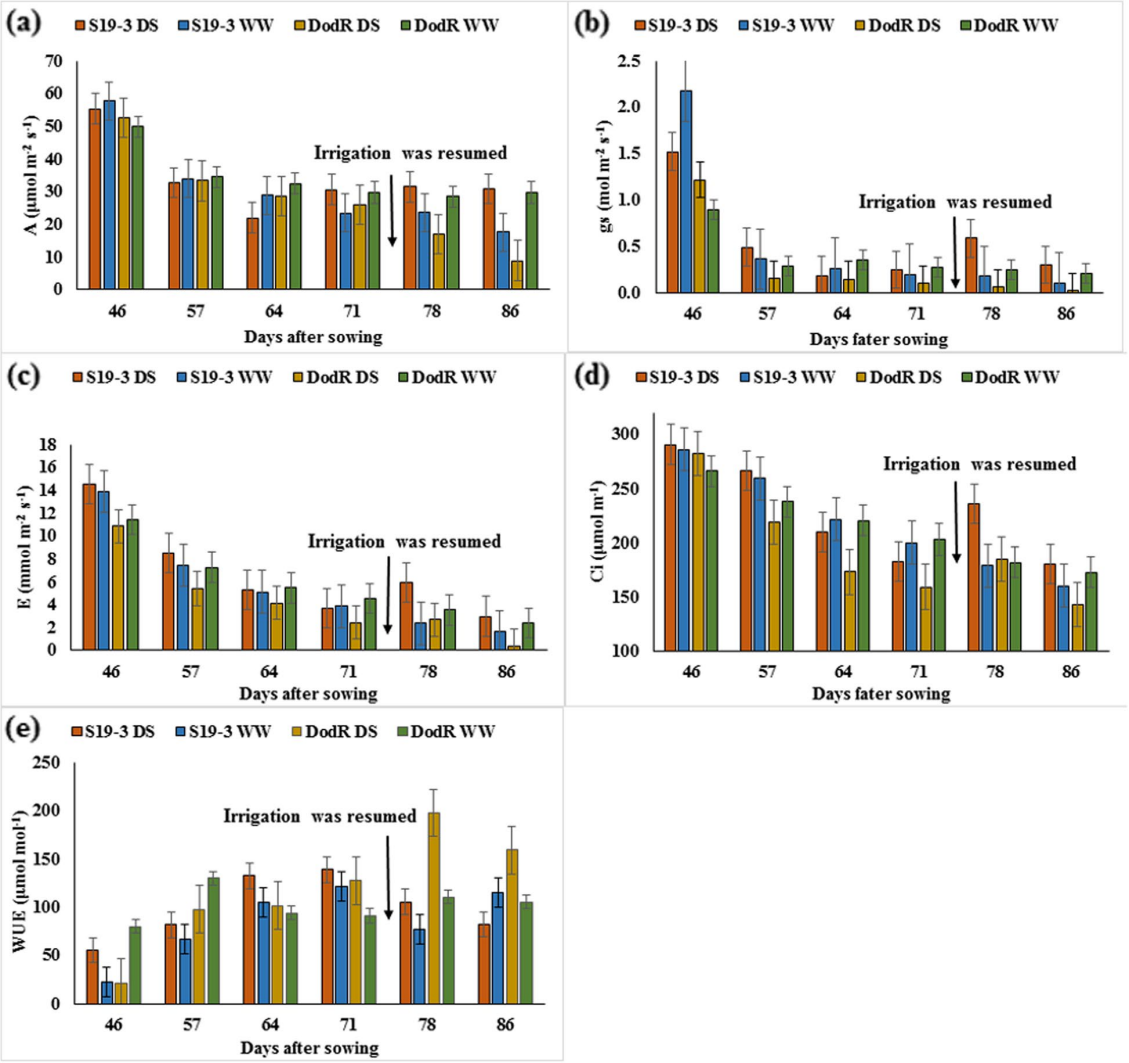
Comparison of (**a**) *photosynthesis rate*, *A* (**b**) *stomatal conductance, gs* (**c**) *transpiration rate, E* (**d**) *intracellular CO*_*2,*_* Ci* and (**E**) water use efficiency, WUE between parental lines, S19-3 and DodR under drought-stressed (DS) and well-watered (WW) conditions. Mean and standard error are indicated at the time of measurement. *n* = 9. Arrow Irrigation was resumed at 74 DAS

Parental lines showed significant differences (*p* < 0.05) for gs and *Ci* at 64 DAS, *E, Ci* and WUE at 71 DAS, *Ci* and WUE at 78 DAS between drought-stressed and well-watered conditions (Fig. 1). S19-3 had significantly higher (*p* < 0.05) *E* at 57 DAS, *A* and *E* at 78 DAS, *gs*, *A*, *E* and *Ci* at 86 DAS but significantly lower (*p* < 0.05) WUE6 compared to DodR under drought-stressed conditions (Fig. 1). The interaction between genotype and environment was significant (*p* < 0.05) for *E* at 57 DAS, A and E at 78 DAS, *gs, A, E, Ci* and WUE at 86 DAS among parental lines (Supplementary Table S1).

**Fig. 2:**
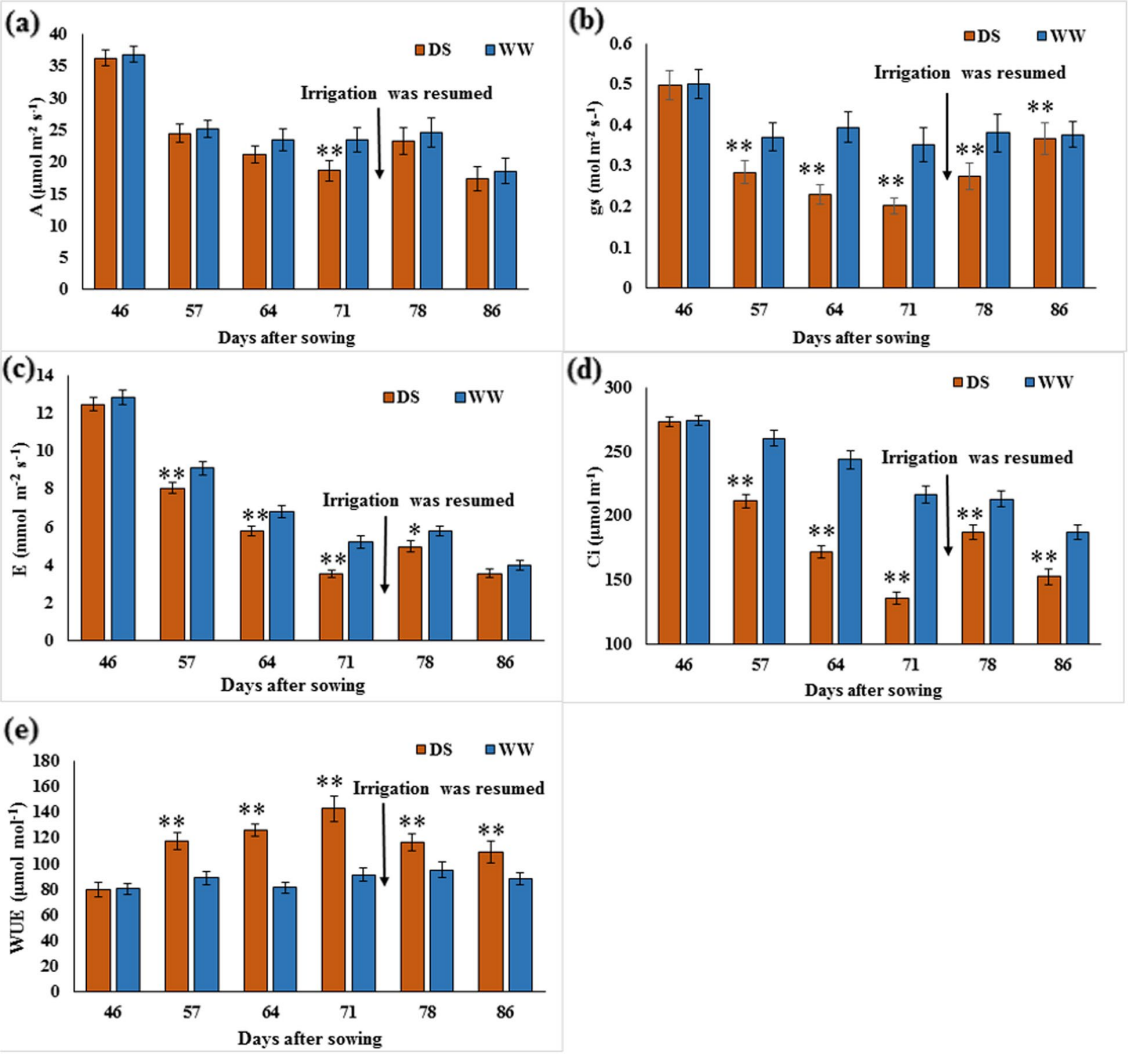
Comparison of (**a**) *photosynthesis rate*, *A* (**b**) *stomatal conductance, gs* (**c**) *transpiration rate, E* (**d**) *intracellular CO*_*2,*_* Ci* and (**e**) water use efficiency, WUE between individual lines under drought-stressed (DS) and well-watered (WW) conditions. Mean and standard error are indicated at the time of measurement. *n* = 36. * = Significant at *p* = 0.05, ** = Significant at *p* = 0.01. Arrow Irrigation was resumed at 74 DAS

On average, *A* declined from 36.24 μmol m^−2^ s^−1^ to 18.61 μmol m^−2^s^−1^ (by 48.6%) was observed under drought-stressed conditions from 47 to 71 DAS followed by recovery to 23.3 μmol m^−2^ s^−1^ (by 25.20%) at 78 DAS after irrigation was resumed, with significant difference (*p* < 0.01) observed between drought-stressed and well-watered treatments at 71 DAS (*p* < 0.01) (Fig. 2a). A significant difference (*p* < 0.05) was observed for *A* among the individual lines during drought period at 64 DAS and 71 DAS and at 78 DAS and 86 DAS after irrigation was resumed. The interaction between the individual lines and water treatment was significant (*p* < 0.05) during drought period at 61 DAS and 71 DAS.

On average, *gs* declined significantly (*p* < 0.01) from 0.497 mol m^−2^ s^−1^ to 0.203 mol m^−2^ s^−1^ (by 59.2%) while WUE increased significantly (*p* < 0.01) from 79.97 μmol mol^−1^ to 142.7 μmol mol^−1^ (by 55.9%) under drought-stressed conditions from 47 to 71 DAS. On average, *gs* then was observed to recover to 0.276 mol m^−2^ s^−1^ (by 35.96%) and WUE to 116.5 μmol mol^−1^ (by 18.36%) at 78 DAS after irrigation was resumed (Fig. 2b and e). A significant difference was observed for *gs* among the individual lines before drought was imposed at 46 DAS (*p* < 0.01), during drought period from 57 to 71 DAS (*p* < 0.01), and at 78 DAS and 86 DAS after irrigation was resumed (*p* < 0.05). WUE exhibited significant difference (*p* < 0.05) among individual lines similar to *gs,* but they were not significantly different during drought period at 64 DAS (*p* = 0.079) and after irrigation was resumed at 86 DAS (*p* = 0.141). Stomatal conductance, *gs* exhibited significant interaction (*p* < 0.05) between individual lines and water conditions before drought conditions was imposed at 46 DAS, during drought period at 57 DAS, 64 DAS and at 78 DAS and 86 DAS after irrigation was resumed. WUE exhibited significant interaction (*p* < 0.05) between individual lines and water conditions similar to *gs,* but not significantly different during drought period at 64 DAS (*p* = 0.051).

Similar to *gs,* on average, *Ci* significantly (*p* < 0.01) declined by 50.37% from 273.6 μmol m^−1^ to 135.8 μmol m^−1^, after drought stress was imposed at 47 DAS and recovered to 186.6 μmol m^−1^ (by 37.41%) at 78 DAS after irrigation was resumed, with significant difference (*p* < 0.01) observed between drought-stressed and well-watered treatments during drought period from 57 to 71 DAS and at 78 DAS and 86 DAS after irrigation was resumed (Fig. 2d). A significant difference was observed for *Ci* among the individual lines during drought period at 71 DAS (*p* < 0.05) and at 78 DAS and 86 DAS after irrigation was resumed (*p* < 0.01). The interaction between individual lines and water conditions for *Ci* was significant (*p* < 0.01) before drought stress was imposed, at 46 DAS and during drought period, at 57 DAS.

On average, *E* significantly (*p* < 0.01) declined from 12.47 mol m^−2^ s^−1^ to 3.54 mol m^−2^ s^−1^, a reduction of 71.61%, under drought-stressed conditions from 46 to 71 DAS and recovered to 4.98 mol m^−2^ s^−1^ (by 40.68%) at 78 DAS after irrigation was resumed, with significant difference observed between drought-stressed and well-watered treatments during drought period from 57 to 71 DAS (*p* < 0.01) and at 78 DAS after irrigation was resumed (*p* < 0.05) (Fig. 2c). A significant difference (*p* < 0.05) for *E* was observed among individual lines and interaction between individual lines and water conditions at 71 DAS.

**Fig. 3:**
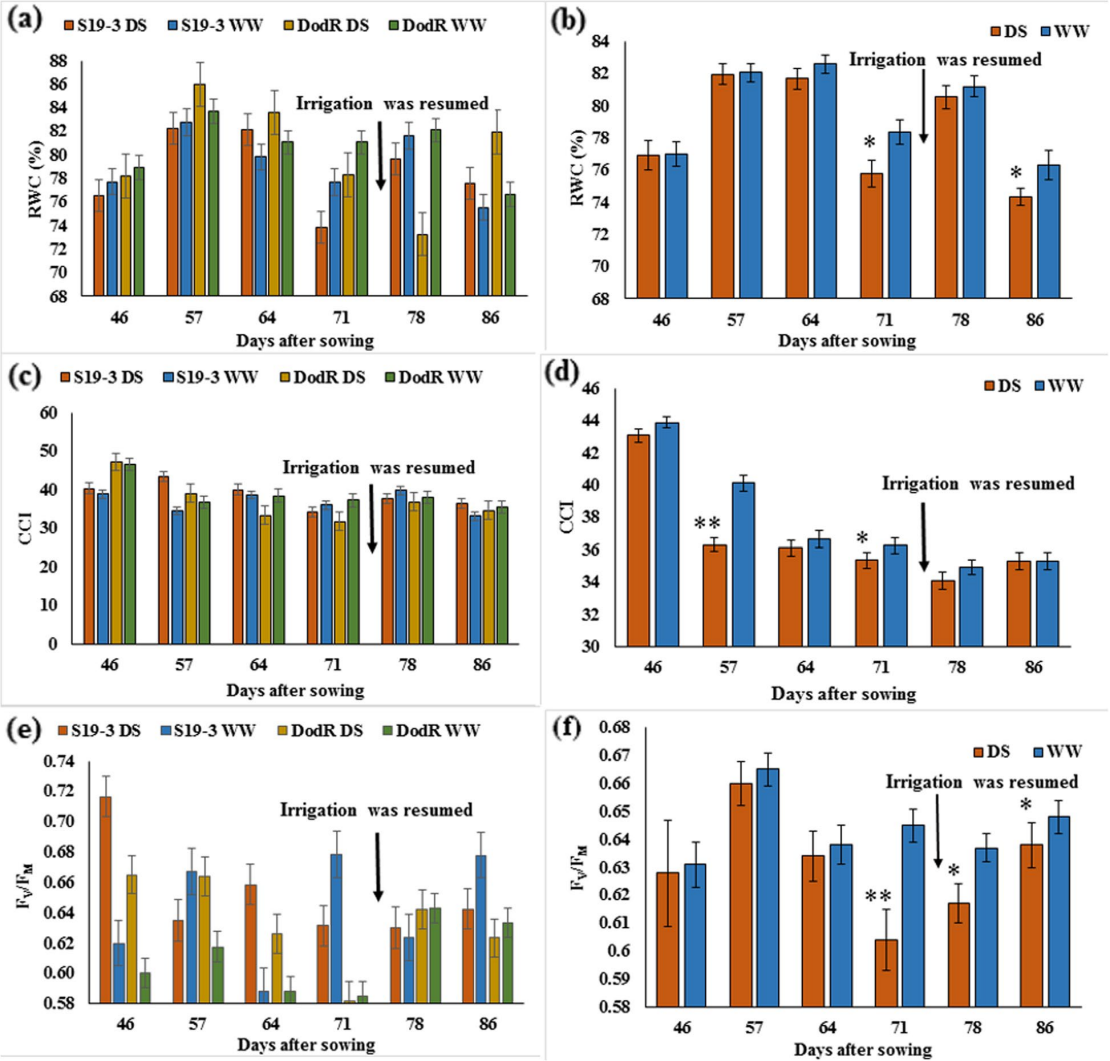
The effect of drought stress on (**a**) and (**b**) *relative water content*, RWC (**c**) and (**d**) *chlorophyll content index,* CCI (**e**) and (**f**) *quantum yield of PSII photochemistry*, *F*_*V*_/*F*_*M*_ in parental lines and the F_4_ segregating population. Data represent mean values ± standard error. DS, drought-stressed conditions; WW, well-watered conditions. * = Significant at *p* = 0.05, ** = Significant at *p* = 0.01. Arrow Irrigation was resumed at 74 DAS

Parental lines showed significant differences (*p* < 0.05) for *F*_*V*_/*F*_*M*_ at 46 DAS and 64 DAS, CCI at 57 DAS and RWC at 78 DAS between drought-stressed and well-watered conditions (Fig. 3). In the F_4_ segregating population, RWC was reduced by 7.55% under drought-stressed conditions from 81.98% at 57 DAS to 75.79% at 71 DAS, with significant difference observed between drought-stressed and well-watered conditions at 71 DAS (*p* < 0.05) and after irrigation was resumed at 86 DAS (*p* < 0.05) (Fig. 3b). A significant difference was observed among the individual lines during drought period at 57 DAS (*p* < 0.01) and after irrigation was resumed at 78 DAS (*p* < 0.05). The interaction between individual lines and water conditions was significant (*p* < 0.05) after drought stress was imposed at 57 DAS.

CCI declined by 17.9% over 28 days (*p* = 0.193) after drought stressed was imposed from 47 to 74 DAS (Fig. 3). CCI under drought-stressed conditions showed 2.5% significant reduction (*p* < 0.05) at 71 DAS compared to well-watered conditions (Fig. 3d). A significant difference was observed among the individual lines during drought period at 71 DAS (*p* < 0.01), after irrigation was resumed at 78 DAS (*p* < 0.05) and 86 DAS (*p* < 0.05). The interaction between individual lines and water conditions was significant (*p* < 0.01) at 71 DAS. *F*_*V*_/*F*_*M*_ declined gradually (*p* = 0.208) by 8.48% during drought period from 0.66 at 57 DAS to 0.60 at 71 DAS (Fig. 3f). *F*_*V*_/*F*_*M*_ had 7.69% significant reduction (*p* < 0.01) under drought-stressed conditions at 71 DAS compared to well-watered conditions (Fig. 3f). An increase in *F*_*V*_/*F*_*M*_ value up to 5.62% was also observed after irrigation was resumed starting from 74 DAS, with significantly higher (*p* < 0.05) *F*_*V*_/*F*_*M*_ observed at 78 DAS and 86 DAS in well-watered treatment. A significant difference was observed among the individual lines before drought conditions was imposed at 46 DAS (*p* < 0.01), during drought period at 64 DAS (*p* < 0.01) and after irrigation was resumed at 78 DAS (*p* < 0.05) and 86 DAS (*p* < 0.05). The interaction between individual lines and water conditions was significant (*p* < 0.05) after drought stress was imposed at 64 and 78 DAS.

### The effect of drought stress on photosynthetic parameters

The average of *A, E, gs, Ci* and WUE, RWC, CCI and *F*_*V*_/*F*_*M*_ under drought-stressed and well-watered conditions during drought period from 47 to 74 DAS were presented in Table 1. The reduction of 1.76% and 9.03% in *A*, 3.26% and 15.23% in *Ci* was observed under drought-stressed conditions in S19-3 and DodR, respectively, compared to well-watered conditions (Table 1).

**Table 1:** Effects of drought stress on photosynthetic parameters under drought stressed (DS) and well-watered (WW) conditions in the F_4_ segregating population derived from S19-3 × DodR and their parental lines

Traits	Treatment	Mean	Min	Max	SD	Variance	Normality	F-probability			S19-3	DodR
								Treatment	Genotypes	G*E		
A (μmol m^−2^ s^−1^)	DS	21.20	6.09	43.46	9.20	84.70	**	*	*	**	29.21	29.32
	WW	24.55	7.12	50.13	11.55	133.50					29.74	32.23
E (mol m^−2^ s^−1^)	DS	5.67	1.79	11.85	10.06	4.02	0.10	**	0.16	0.27	5.85	4.02
	WW	7.14	1.42	15.06	13.64	7.32					5.48	5.60
gs (mol m^−2^ s^−1^)	DS	0.25	0.09	0.79	0.7.02	0.02	**	**	**	0.15	0.32	0.12
	WW	0.48	0.09	0.80	7.91	0.95					0.27	0.29
Ci (μmol m^−1^)	DS	175.40	97.15	249.70	152.50	1188.00	**	**	0.12	0.25	219.58	186.85
	WW	238.20	120.90	524.60	403.70	2643.00					226.98	220.40
WUE (μmol mol^−1^)	DS	129.40	56.04	260.20	204.20	1318.00	**	**	**	**	101.99	104.59
	WW	88.87	37.57	152.10	124.60	559.00					86.29	99.99
RWC (%)	DS	79.57	70.21	87.54	17.33	15.54	0.22	*	0.46	0.83	79.04	82.13
	WW	80.85	71.92	88.42	16.50	13.48					80.36	81.95
CCI	DS	38.05	27.73	53.25	25.52	25.23	0.96	0.09	*	*	38.57	35.19
	WW	36.26	20.20	52.10	31.90	36.69					36.37	37.46
*F*_*V*_/*F*_*M*_	DS	0.64	0.43	0.77	0.34	0.00	**	0.43	0.08	0.11	0.64	0.62
	WW	0.64	0.53	0.73	0.20	0.00					0.64	0.61

*A* Photosynthesis rate, *gs* Stomatal conductance, *E* Transpiration rate, *Ci* Intracellular CO_2_, *WUE* Water use efficiency, *RWC* Relative water content, *CCI* Chlorophyll content index, *F*_*V*_/*F*_*M*_ Quantum yield of PSII photochemistry. *SD* Standard deviation, G*E Interaction between conditions and genotypes, * = Significant at *p* = 0.05, ** = Significant at *p* = 0.01

A significant reduction (*p* < 0.05) of 13.65% in *A*, 20.59% in *E*, 47.92% in *gs*, 26.36% in *Ci* and 1.58% in RWC was observed in the individual lines under drought-stressed conditions compared to well-watered conditions (Table 1). Compared to well-watered conditions, WUE significantly (*p* < 0.01) increased by 45.61% under drought-stressed conditions (Table 1). *A, gs,* WUE and CCI showed significant difference (*p* < 0.05) among individual lines. The interaction between conditions and individual lines was significant (*p* < 0.05) for *A*, WUE and CCI.

*A* positively correlated with *gs* (r_WW_ = 0.55, *p* < 0.01; r_DS_ = 0.53,* p* < 0.01), *Ci* (r_WW_ = 0.66, *p* < 0.01; r_DS_ = 0.46, *p* < 0.05) and *E* (r_WW_ = 0.60, *p* < 0.05; r_DS_ = 0.35, *p* = 0.07), and negatively correlated with RWC (r_WW_ = –0.45, *p* < 0.05; r_DS_ = –0.07, *p* = 0.73) (Supplementary Table S2). *gs* positively correlated with *Ci* (r_WW_ = 0.49, *p* < 0.05; r_DS_ = 0.32, *p* = 0.09) and* E* (r_WW_ = 0.41, *p* < 0.05; r_DS_ = 0.17, *p* = 0.53), and negatively correlated with WUE (r_WW_ = –0.71, *p* < 0.01; r_DS_ = –0.65, *p* < 0.01) (Supplementary Table S2).

### Detection of QTLs associated with photosynthetic parameters under drought-stressed and well-watered conditions

**Fig. 4:**
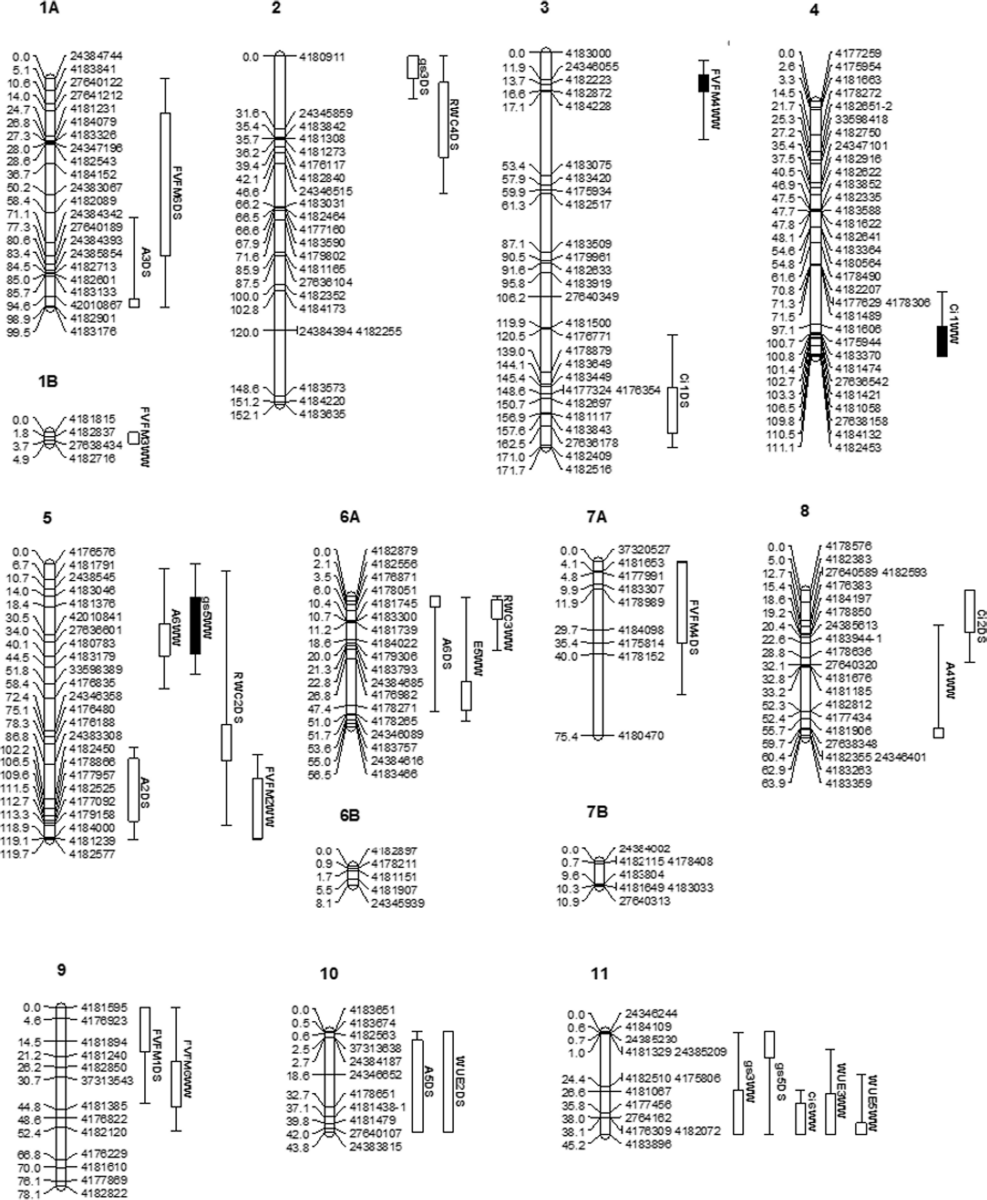
Map position of the quantitative trait loci (QTL) under drought-stressed (DS) and well-watered (WW) conditions in the F_4_ segregating population developed from S19-3 × DodR. Rectangular bars represent the 1- and 2-LOD QTL interval (inner and outer interval). Solid rectangular bars represent significant QTLs, while blank bars represent putative QTLs. LG1, LG6 and LG7 were divided into subgroups ‘1A’ and ‘1B’, respectively, based on the association observed in the maximum likelihood mapping (MLM) due to insufficient linkage to complete the map using regression mapping (RM).* A* Photosynthesis rate, *gs* Stomatal conductance*, E* Transpiration rate*, Ci* Intracellular CO_2_*, WUE* Water use efficiency*, RWC* Relative water content, *F*_*V*_*/F*_*M*_ Quantum yield of PSII photochemistry

Significant and putative QTLs for photosynthetic traits were detected under both water regimes in the F_4_ segregating population (Fig. 4). Most QTLs were distributed in LG5, LG6A and LG11. Significant QTL for *gs5* (LOD: 3.09, 37.7% of the PVE) and significant QTL for F_V_/F_M_4 (LOD: 3.06, 36.5% of the PVE) under well-watered conditions were mapped on LG5 and LG3, respectively (Table 2). Putative QTL for *gs3* (LOD: 2.12, 26.6% of the PVE) and putative QTL for WUE3 (LOD: 2.26, 28.7% of the PVE) under well-watered conditions were co-located on LG11 (35.83 cM, nearest marker: 4,177,456) with overlapping confidence intervals (Table 2). Putative QTL for *Ci6* (LOD: 2.41, 29.8% of the PVE) and putative QTL for WUE5 (LOD: 2.39, 33.5% of the PVE) under well-watered conditions were co-located on LG11 (45.18 cM, nearest marker: 4,183,896) with overlapping confidence intervals (Table 2). Five clustered QTLs were found to have overlapping confidence intervals for photosynthetic traits, which included *gs5* and *A6* under well-watered conditions, *A2* under drought-stressed conditions and F_V_/F_M_2 under well-watered conditions on LG5, *A6* under drought-stressed conditions and RWC3 under well-watered conditions on LG6A, *A5* and WUE2 under drought-stressed conditions on LG10, *gs3,* WUE3, *Ci6* and WUE5 under well-watered conditions on LG11 (Fig. 4).

**Table 2:** Significant and putative QTLs for photosynthetic traits under drought-stressed (DS) and well-watered (WW) conditions in the F_4_ segregating population derived from S19-3 × DodR

Traits	Treatment	GW LOD	IM LOD	Group	Position	Locus	PVE	Additive Effect
A2	DS	2.80	2.70	5	106.49	4,178,866	29.60	–5.89
A3	DS	3.00	2.01	1A	99.53	4,183,176	25.70	–6.66
A4	WW	2.90	2.19	8	60.37	4,182,355	27.00	–10.80
A5	DS	2.90	2.27	10	32.66	4,178,651	29.50	–9.07
A6	DS	2.90	2.11	6A	0.00	4,182,879	28.50	–8.52
A6	WW	2.90	2.15	5	33.96	27,636,601	28.90	8.38
E5	WW	2.90	2.08	6A	47.37	4,178,271	27.40	1.64
gs3	DS	3.00	2.47	2	0.00	4,180,911	37.80	–0.08
gs3	WW	3.00	2.12	11	35.83	4,177,456	26.60	–0.18
gs5	DS	2.90	2.04	11	0.00	24,346,244	32.40	–0.18
gs5	WW	3.00	3.09	5	18.35	4,181,376	37.70	0.24
Ci1	DS	2.90	2.19	3	148.57	4,177,324	25.00	16.26
Ci1	WW	3.00	2.52	4	103.26	4,181,421	27.60	17.15
Ci2	DS	2.90	2.06	8	12.68	27,640,589	26.00	–21.27
Ci6	WW	2.90	2.41	11	45.18	4,183,896	29.80	–37.69
WUE2	DS	2.80	1.85	10	42.02	27,640,107	26.00	28.14
WUE3	WW	2.90	2.26	11	35.83	4,177,456	28.70	20.14
WUE5	WW	2.90	2.39	11	45.18	4,183,896	33.50	33.33
RWC2	DS	2.90	2.01	5	75.12	4,176,480	26.50	3.77
RWC3	WW	3.10	2.14	6A	5.99	4,178,051	27.90	–1.95
RWC4	DS	2.90	2.33	2	36.15	4,181,273	29.20	–3.61
*F*_*V*_/*F*_*M*_	DS	3.10	2.58	9	14.50	4,181,894	42.50	0.09
F_V_/F_M_2	WW	3.00	2.73	5	112.71	4,177,092	33.40	–0.03
F_V_/F_M_3	WW	3.00	2.05	1B	1.85	4,182,837	27.00	0.03
F_V_/F_M_4	DS	3.00	2.59	7A	4.77	4,177,991	27.20	–0.05
F_V_/F_M_4	WW	3.00	3.06	3	13.72	4,182,223	36.50	0.03
F_V_/F_M_6	DS	3.00	2.55	1A	10.61	27,640,122	32.40	0.03
F_V_/F_M_6	WW	3.00	2.26	9	30.66	37,313,543	31.10	–0.03

*GW LOD* Genome-Wide logarithm of odds, *IM LOD* Interval mapping logarithm of odds, *PVE* Phenotypic variation explanation. *A* Photosynthesis rate, *gs* Stomatal conductance, *E* Transpiration rate, *Ci* Intracellular CO_2_, *WUE* Water use efficiency, *RWC* Relative water content, *F*_*V*_/*F*_*M*_ Quantum yield of PSII photochemistry

## Discussion

Stomatal closure usually happened during the initial stages of drought stress, which results in the reduction of transpiration in plant leaves, a decrease in CO_2_ flow into leaves, a decline in net photosynthesis, and ultimately reduced plant growth [14, 26, 27]. In the present study, WUE, calculated as *A/gs*, increased after drought stress was imposed, then declined gradually after irrigation was resumed under drought-stressed conditions in the F_4_ segregating population. Singh and Reddy [28] reported that WUE increased under drought stress in 15 cowpea genotypes, suggesting that stomatal regulation was a major limitation to photosynthesis and plant growth. WUE is regulated by *gs* and multiple factors including the available energy impinging on the leaf, vapour pressure deficit, and aerodynamic exchange [29]. The negative correlation between WUE and *gs* under well-watered conditions (*r* = –0.79, *p* < 0.05) and under drought-stressed conditions (*r* = –0.63, *p* < 0.01) suggests that *gs* decreases faster than *A*, leading to increased WUE under drought stress [30]. The QTL associated with *gs*3 and WUE3 were mapped on LG11 with overlapping confidence intervals in the F_4_ population, which may suggest that these traits are controlled by the same loci. Similar findings have been reported in Sorghum that the QTL for *gs* was associated with reduced E and increased WUE in Sorghum [24].

Genotypes with high *stomatal conductance* and WUE in response to drought stress were suggested to have good drought tolerance and adaptation ability [28]. For example, a drought-tolerant cowpea cultivar (PO) maintained higher photochemical activity and leaf gas exchange under water deficit and showed faster recovery of photosynthesis after irrigation was resumed than the drought-sensitive cultivar (SI), revealing possible mechanisms enabling plants to overcome stressful conditions [31, 32]. Plants maintain high water status by reducing stomatal conductance during periods of drought stress, which involves either drought avoidance or tolerance or both mechanisms [30, 33, 34]. The positive correlation between *A* and *Ci* under well-watered conditions (*r* = 0.67, *p* < 0.05) and under drought-stressed conditions (*r* = 0.42, *p* < 0.05) suggests that lower internal CO_2_ accumulation concentration during drought is responsible for the reduction in photosynthesis [35]. In the present study, the QTL for *Ci6* and WUE5 were co-located on LG11 with overlapping confidence intervals, which may suggest that these traits are controlled by the same loci.

Chlorophyll content and *F*_*V*_/*F*_*M*_ are non-stomatal limiting factors and capture light energy for plant photosynthesis [30, 36]. In the present study, drought stress significantly reduced (*p* < 0.05) CCI and *F*_*V*_/*F*_*M*_ in the F_4_ segregating population, which suggests that the ability of bambara groundnut plants to capture light energy for plant photosynthesis is significantly curtailed by drought conditions. Similar to these findings, Mafakheri et al. [26] reported that drought significantly reduced total chlorophyll content (*p* < 0.05) under drought stress during vegetative growth in three chickpea cultivars. Rahbarian et al. [37] also reported that drought stress reduced *F*_*V*_/*F*_*M*_ in two drought-tolerant genotypes and two drought-sensitive genotypes of Chickpea. Additionally, in a study involving several bambara groundnut landraces, CCI was lower under water-deficit compared to irrigated conditions [14, 38]. The *F*_*V*_/*F*_*M*_ value was also reported to have declined by 25% at the end of drought stress trial involving three bambara groundnut landraces [39]. Significant QTL associated with F_V_/F_M_4 under well-watered conditions was mapped on LG3 with 36.5% of the PVE, while putative QTL for F_V_/F_M_4 was mapped on LG7A with reduced PVE (27.2 of the PVE) under drought-stressed conditions in the F_4_ population. Similar to *gs5*, a reduced PVE was detected under drought-stressed conditions compared to well-watered conditions, suggesting the traits identified under well-watered conditions were unable to fully express their potential trait values under drought conditions [22].

RWC is an indicator of plant water status revealing the stress intensity [40]. In the present study, RWC increased in the initial stage of drought, declined gradually until the end of drought period, suggesting some individual lines have the ability to adapt to drought stress. RWC was reported to have decreased by 21% – 24% with time after water deficit and increased by 13% – 17% after irrigation was resumed in bambara groundnut [39]. RWC was higher in well-watered plants than drought-stressed plants, although bambara groundnut accessions were still able to maintain high RWC despite the water stress [41]. Keyvan [35] reported wheat cultivars with high RWC under drought stress conditions to be resistant. The putative QTL for RWC3 and *seeds weight per plant* under well-watered conditions were mapped on LG6A with overlapping confidence intervals, suggesting RWC and seed yield may be controlled by the same gene [22]. Further validation of consensus markers, significant QTLs associated with various traits and candidate genes is required in different populations, across locations and seasons in bambara groudnnut. Individual lines with overall superior performance such as high *gs, E*, RWC, *F*_*V*_/*F*_*M*_ and CCI than S19-3 under drought-stressed conditions are recommended for further field investigation to develop drought-tolerant varieties (Supplementary Table S3). The major QTLs identified in this study are essential to support the development of improved varieties of bambara groundnut in molecular-enabled breeding programmes.

## Conclusions

The development of drought resistant materials is essential to cope with the effects of climate change, especially in the tropical arid and semi-arid areas where rainfall is scarce and erratic. Drought stress significantly reduced (*p* < 0.05) *gs, A, E, Ci* and RWC, while WUE significantly increased (*p* < 0.01) under drought-stressed conditions in the F_4_ segregating population. The linear correlation between photosynthetic parameters suggests the synergy of photosynthesis mechanisms when plant response to drought stress. Significant QTL for *gs* and *F*_*V*_/*F*_*M*_ under well-watered conditions were mapped on LG5 and LG3, respectively. QTLs identified under well-watered conditions would reflect the intrinsic genetic mechanisms underlying photosynthetic parameters. Five clustered QTLs were found to have overlapping confidence intervals for photosynthetic traits, which included *gs, A, Ci,* WUE, *F*_*V*_/*F*_*M*_ and RWC under well-watered conditions and *A* and WUE under drought-stressed conditions, suggesting these traits are controlled by the same major QTLs. The QTLs identified in this study are essential to identify candidate genes related to photosynthetic traits in response to drought stress in bambara groundnut.

## Methods

### Plant material and experimental design 

A total of 36 individual lines of the F_4_ segregating population derived from a cross between S19-3 and DodR were evaluated in a rainout shelter at the University of Nottingham Malaysia (2°56′46.74"N; 101°52′24.35"E) with mean air temperature of 36 °C/25 °C day/night and relative humidity of 58%/91% day/night from April to July 2019. The experiment was carried out in a completely randomized design (CRD) with three replicates and two treatments, drought-stressed and well-watered treatments [22]. Each of the replicates was represented by one plant from each of the individual lines. Irrigation for the well-watered conditions was continued throughout the experiment while the drought-stressed conditions was imposed after 100% flowering was observed at 47 days after sowing (DAS) and no further irrigation was applied until early pod-filling stage at 74 DAS, at which irrigation of plants for the drought-stressed conditions was resumed [22].

### Field management

A trickle irrigation system was set to irrigate the plants at 07:00 and 19:00 h for 10 min with a flow rate of 2 L/h, with each tube 6 m in length [22]. A distance of 40 cm × 30 cm was kept between the plants. NPK (nitrogen, phosphorus and potassium) fertiliser was applied at a rate of 20:40:60 kg/ha (133 kg/ha NPK (15:15:15), 44 kg/ha TSP (triple-super-phosphate) and 67 kg/ha MOP (muriate of potash) at sowing and after emergence [22]. All other agronomic procedures, such as weeding and spraying of pesticides, were carried out when necessary [22].

### Soil moisture content

Two evenly spaced PR2 profile tubes (Delta-T Devices Ltd., Cambridge, UK) were inserted into the centre of each of each plot with a distance of 3 m between two profile tubes in each plot [22]. There were 12 access tubes in total [22]. Three PR2 readings %Vol (volumetric water content as a percentage) were taken twice a week between 0900 and 1100 h at soil depth of 100, 200, 300, 400, 600 and 1000 mm from seeds sowing until maturity [22].

### Photosynthetic parameters

*A, gs, Ci* and *E* were measured by LI-6400XT Portable Photosynthesis System (Li-Cor, Lincoln, USA). *Water use efficiency* (WUE) was estimated as the ratio of A/gs [28].

*Relative water content* (RWC) was calculated as:

$$\mathrm{RWC }= [(\mathrm{Fw}-\mathrm{Dw}) / (\mathrm{Tw}-\mathrm{Dw})] \times 100$$

where FW = fresh weight of leaves, TW = turgid weight of leaves after incubating leaves in distilled water for 24 h, and DW = dry weight of leaves after oven drying at 80 °C for 48 h.

CCI was measured by chlorophyll meter SPAD-502 (Spectrum Technologies, Inc., Aurora, Illinois, USA). Three readings were taken per leaf, three leaves per plant, and averaged to give a final reading. Quantum yield of PSII photochemistry (*F*_*V*_/*F*_*M*_) was estimated from dark-adapted leaves for 30 min using FlourPen FP 100 (PSI, CZ, Czech Republic). Photosystem II quantum yield is equivalent to ratio of variable fluorescence/maximal fluorescence (*F*_*V*_/*F*_*M*_) in dark-adapted samples.

Readings were taken for *A, gs, E, Ci,* RWC, CCI and *F*_*V*_/*F*_*M*_ on the middle leaflet of one most fully expanded leaf between 08:00 and 12:00 h for all individual lines, starting from 50% flowering observed at 46 DAS before drought conditions was imposed, during drought period at 57 DAS, 64 DAS and 71 DAS, and after irrigation was resumed, at 78 DAS and 86 DAS.

### Genetic linkage map construction and QTL analysis

The genetic linkage map was constructed using an F_2_ individual population derived from the same parents [22]. Genetic linkage map and phenotypic data from drought-stressed and well-watered conditions were subjected to QTL analysis using MapQTL 6.0 software [42]. The significant threshold of the Genome-Wide (GM) LOD threshold was obtained from permutation test using 10,000 repetitions at *p* < 0.05 (5%). Interval mapping (IM) was carried out following the permutation test and the LOD values from IM was compared with GW LOD threshold at *p* < 0.05 from the permutation test. Significant QTLs were detected if the LOD score was equivalent or higher than GM LOD threshold. Putative QTLs were detected if the LOD score was lower than GM LOD threshold by up to a 1-LOD interval [20, 22]. MapChart 2.3.2 [43] was used to depict the linkage groups and QTLs. QTLs explaining more than 20.0% of the phenotypic variation (occurring at least once) or more than 10% of the phenotypic variation (occurring at least twice) were defined as major QTL, whereas QTL was defined as minor QTL [44, 45].

### Data analysis

Normality of trait data was examined using Shapiro–Wilk normality test and data transformation was performed for non-normally distributed trait data. Two-way analysis of variance (ANOVA) and Pearson’s correlation coefficient analysis were conducted for all photosynthetic parameters using 18^th^ edition of Genstat Statistical package (18th edition, VSN International, UK).

## Supplementary Information

**Additional file 1: Supplementary Table S1.** The effects of drought stress on photosynthetic parameters under drought-stressed (DS) and well-watered (WW) conditions in parental lines, S19-3 and DodR.

**Additional file 2: Supplementary Table S2.** Correlation coefficient analysis of photosynthetic parameters under drought-stressed and well-watered conditions in the F4 segregating populations of bambara groundnut derived from S19-3 × DodR.

**Additional file 3: Supplementary Table S3.** Potential with superior performance than S19-3 for advancement based on photosynthesis rate, stomatal conductance, transpiration rate, intracellular CO_2_, water use efficient, relative water content, chlorophyll content index and quantum yield of PSII photochemistry in the F_4_ segregating population derived from S19-3 × DodR.

**Additional file 4: Supplementary Fig S1.** Soil moisture content measurements at depth 100 mm, 200 mm and 300 mm based on PR2 reading (% vol) under drought-stressed (DS) well-watered (WW) conditions. Data represent mean values of soil moisture content during plant growth season in 2019; *n* = 6. Data represent mean values ± standard error. (Gao et al. 2022 [22]).

**Additional file 5: Supplementary Fig S2.** Soil moisture content measurements at depth of 400 mm, 600 mm and 1000 mm based on PR2 reading (% vol) under drought stress (DS) conditions plots and well-watered (WW) conditions plots. Data represent mean values of soil moisture content during plant growth season in 2019; *n* = 6. Data represent mean values ± standard error (Gao et al. 2022 [22]).

## Data Availability

The datasets generated during and/or analysed during the current study are available from the corresponding author on reasonable request.

## Abbreviation

A: Photosynthesis rate
A2: Photosynthesis rate at 57 days after sowing
A3: Photosynthesis rate at 64 days after sowing
A4: Photosynthesis rate at 71 days after sowing
A5: Photosynthesis rate at 78 days after sowing
A6: Photosynthesis rate at 86 days after sowing
ANOVA: Analysis of variance
CCI: Chlorophyll content index
Ci: Intracellular CO_2_
Ci1: Intracellular CO_2_ at 46 days after sowing
Ci2: Intracellular CO_2_ at 57 days after sowing
Ci6: Intracellular CO_2_ at 86 days after sowing
CRD: Completely randomized design
DArTseq-based SNP markers: Diversity array technology sequencing based single nucleotide polymorphism markers
DAS: Days after sowing
DS: Drought-stressed
E: Transpiration rate
E5: Transpiration rate at 78 days after sowing
*F*_*V*_/*F*_*M*_: Quantum yield of PSII photochemistry
F_V_/F_M_1: Quantum yield of PSII photochemistry at 46 days after sowing
F_V_/F_M_2: Quantum yield of PSII photochemistry at 57 days after sowing
F_V_/F_M_3: Quantum yield of PSII photochemistry at 64 days after sowing
F_V_/F_M_4: Quantum yield of PSII photochemistry at 71 days after sowing
F_V_/F_M_6: Quantum yield of PSII photochemistry at 86 days after sowing
G*E: Interaction between conditions and genotypes
gs: Stomatal conductance
gs3: Stomatal conductance at 64 days after sowing
gs5: Stomatal conductance at 78 days after sowing
GW LOD: Genome-Wide logarithm of odds
IM LOD: Interval mapping logarithm of odds
MLM: Maximum likelihood mapping
MOP: Muriate of potash
NPK: Nitrogen, phosphorus and potassium
PVE: Phenotypic variation explanation
QTL: Quantitative trait loci
RM: Regression mapping
RWC: Relative water content
RWC2: Relative water content at 57 days after sowing
RWC3: Relative water content at 64 days after sowing
RWC4: Relative water content at 71 days after sowing
SD: Standard deviation
TSP: Triple-super-phosphate
WUE: Water use efficiency
WUE2: Water use efficiency at 57 days after sowing
WUE3: Water use efficiency at 64 days after sowing
WUE5: Water use efficiency at 78 days after sowing
WW: Well-watered
