# Supplementary material for: Deciphering the molecular basis for photosynthetic parameters in Bambara groundnut (Vigna subterranea L. Verdc) under drought stress
Source: BMC Plant Biol. 2023 May 30;23:287. doi: 10.1186/s12870-023-04293-w (PMC10228090; doi:10.1186/s12870-023-04293-w)
Supplement: Supplementary file 1 — Additional file 1: Supplementary Table S1. The effects of drought stress on photosynthetic parameters under drought-stressed (DS) and well-watered (WW) conditions in parental lines, S19-3 and DodR. [file 12870_2023_4293_MOESM1_ESM.pdf]

Deciphering the molecular basis for photosynthetic parameters in Bambara groundnut (*Vigna subterranea* L. Verdc) under drought stress

Xiuqing Gao[1,2*], Hui Hui Chai[2], Wai Kuan Ho[2], Sean Mayes[3,4] and Festo Massawe[2*]

[1]School of Chemistry and Chemical Engineering, North University of China, Taiyuan 030051, China
[2]Future Food Beacon, School of Biosciences, University of Nottingham Malaysia, Jalan Broga, Semenyih 43500, Selangor Darul Ehsan, Malaysia
[3] Plant and Crop Sciences, School of Biosciences, University of Nottingham, Sutton Bonington Campus, Leics, Loughborough LE12 5RD, UK
[4] Crops for the Future (UK) CIC 76-80 Baddow Road, Chelmsford, Essex CM2 7PJ, UK
* Correspondence: Xiuqing Gao, ORCID ID: 0000-0002-9056-6854, 20220061@nuc.edu.cn and Festo Massawe, festo.massawe@nottingham.edu.my, ORCID ID: 0000-0002-0744-4777

Supplementary Table S1 The effects of drought stress on photosynthetic parameters under drought-stressed (DS) and well-watered (WW) conditions in parental lines, S19-3 and DodR.

Traits	Treatment	S19-3		DodR		Fpr		
		Min	Max	Min	Max	Treatment	Genotypes	G*E
A	DS	18.22	37.91	21.39	37.25	0.14	0.80	0.67
	WW	21.42	48.07	22.66	43.48			
E	DS	2.91	8.22	1.63	5.44	0.11	*	*
	WW	4.14	7.73	3.75	8.16			
gs	DS	0.11	0.50	0.01	0.34	0.20	0.08	0.05
	WW	0.06	0.53	0.01	0.63			
Ci	DS	192.22	241.47	140.62	218.37	0.06	*	0.22
	WW	188.11	270.48	157.10	249.51			
WUE	DS	3.73	7.55	4.97	7.06	0.23	0.25	0.88
	WW	3.79	7.12	4.15	7.68			
RWC	DS	71.73	82.12	76.91	87.16	0.76	0.09	0.58
	WW	75.75	87.59	77.15	86.67			
CCI	DS	26.90	51.07	28.37	41.80	0.98	0.54	0.30
	WW	23.27	45.67	27.33	44.37			
F_V/F_M	DS	0.57	0.70	0.56	0.69	0.56	0.09	0.59
	WW	0.57	0.72	0.48	0.68			

Note: A Photosynthesis rate, *gs* Stomatal conductance*, E* Transpiration rate*, Ci* Intracellular CO_2*, WUE* Water use efficiency, *RWC* Relative water content, *CCI* Chlorophyll content index, F_V/F_M Quantum yield of PSII photochemistry, * = Significant at $p = 0.05$, *G*E* interaction between conditions and genotypes.
