# Supplementary material for: Deciphering the molecular basis for photosynthetic parameters in Bambara groundnut (Vigna subterranea L. Verdc) under drought stress
Source: BMC Plant Biol. 2023 May 30;23:287. doi: 10.1186/s12870-023-04293-w (PMC10228090; doi:10.1186/s12870-023-04293-w)
Supplement: Supplementary file 2 — Additional file 2: Supplementary Table S2. Correlation coefficient analysis of photosynthetic parameters under drought-stressed and well-watered conditions in the F4 segregating populations of bambara groundnut derived from S19-3 × DodR. [file 12870_2023_4293_MOESM2_ESM.pdf]

Deciphering the molecular basis for photosynthetic parameters in Bambara groundnut (*Vigna subterranea* L. Verdc) under drought stress

Xiuqing Gao[1,2*], Hui Hui Chai[2], Wai Kuan Ho[2], Sean Mayes[3,4] and Festo Massawe[2*]

[1]School of Chemistry and Chemical Engineering, North University of China, Taiyuan 030051, China
[2]Future Food Beacon, School of Biosciences, University of Nottingham Malaysia, Jalan Broga, Semenyih 43500, Selangor Darul Ehsan, Malaysia
[3] Plant and Crop Sciences, School of Biosciences, University of Nottingham, Sutton Bonington Campus, Leics, Loughborough LE12 5RD, UK
[4] Crops for the Future (UK) CIC 76-80 Baddow Road, Chelmsford, Essex CM2 7PJ, UK
* Correspondence: Xiuqing Gao, ORCID ID: 0000-0002-9056-6854, 20220061@nuc.edu.cn and Festo Massawe, festo.massawe@nottingham.edu.my, ORCID ID: 0000-0002-0744-4777

Supplementary Table S2 Correlation coefficient analysis of photosynthetic parameters under drought-stressed and well-watered conditions in the F_4 segregating populations of bambara groundnut derived from S19-3 × DodR.

	A	gs	Ci	E	WUE	RWC	CCI	F_V/F_M
A	-	**0.53****	**0.46***	0.35	–0.24	–0.07	–0.29	–0.09
gs	**0.55****	-	0.32	0.13	**–0.65****	–0.22	–0.16	–0.23
Ci	**0.66****	**0.49***	-	0.17	–0.31	0.14	–0.24	0.22
E	**0.60****	**0.41***	**0.56****	-	–0.14	–0.18	0.12	0.02
WUE	–0.15	**–0.71****	–0.31	–0.28	-	0.30	0.03	0.19
RWC	**–0.45***	–0.21	–0.19	–0.35	0.08	-	0.36	0.28
CCI	–0.25	–0.30	–0.37	–0.12	0.15	–0.24	-	**0.44***
F_V/F_M	–0.01	–0.01	–0.15	–0.06	0.22	0.33	–0.24	-

A Photosynthesis rate, *gs* Stomatal conductance*, E* Transpiration rate*, Ci* Intracellular CO_2*, WUE* Water use efficiency, *RWC* Relative water content, *CCI* Chlorophyll content index, F_V/F_M Quantum yield of PSII photochemistry, * = Significant at $p = 0.05$, ** = Significant at $p = 0.01$, Values below diagonal are correlation coefficients among traits under well-watered conditions; values above diagonal are correlation coefficients among traits under drought-stressed conditions.
