# Supplementary material for: Deciphering the molecular basis for photosynthetic parameters in Bambara groundnut (Vigna subterranea L. Verdc) under drought stress
Source: BMC Plant Biol. 2023 May 30;23:287. doi: 10.1186/s12870-023-04293-w (PMC10228090; doi:10.1186/s12870-023-04293-w)
Supplement: Supplementary file 3 — Additional file 3: Supplementary Table S3. Potential with superior performance than S19-3 for advancement based on photosynthesis rate, stomatal conductance, transpiration rate, intracellular CO2, water use efficient, relative water content, chlorophyll content index and quantum yield of PSII photochemistry in the F4 segregating population derived from S19-3 × DodR. [file 12870_2023_4293_MOESM3_ESM.pdf]

# Deciphering the molecular basis for photosynthetic parameters in Bambara groundnut (*Vigna subterranea* L. Verdc) under drought stress

Xiuqing Gao[1,2*], Hui Hui Chai[2], Wai Kuan Ho[2], Sean Mayes[3,4] and Festo Massawe[2*]

[1]School of Chemistry and Chemical Engineering, North University of China, Taiyuan 030051, China
[2]Future Food Beacon, School of Biosciences, University of Nottingham Malaysia, Jalan Broga, Semenyih 43500, Selangor Darul Ehsan, Malaysia
[3] Plant and Crop Sciences, School of Biosciences, University of Nottingham, Sutton Bonington Campus, Leics, Loughborough LE12 5RD, UK
[4] Crops for the Future (UK) CIC 76-80 Baddow Road, Chelmsford, Essex CM2 7PJ, UK
* Correspondence: Xiuqing Gao, ORCID ID: 0000-0002-9056-6854, 20220061@nuc.edu.cn and Festo Massawe, festo.massawe@nottingham.edu.my, ORCID ID: 0000-0002-0744-4777

Supplementary Table S3 Potential with superior performance than S19-3 for advancement based on photosynthesis rate, stomatal conductance, transpiration rate, intracellular $CO_2$, water use efficiency, relative water content, chlorophyll content index and quantum yield of PSII photochemistry in the $F_4$ segregating population derived from S19-3 × DodR.

| Genotype | Treatment | A (µmol $m^{-2}$ $s^{-1}$) | E (mol $m^{-2}s^{-1}$) | gs (mol $m^{-2}$ $s^{-1}$) | Ci (µmol $m^{-1}$) | WUE (µmol $mol^{-1}$) | RWC (%) | CCI | $F_V/F_M$ |
|---|---|---|---|---|---|---|---|---|---|
| Line-1 | DS | 22.91 | 7.82 | 0.16 | 148.15 | 141.17 | 80.07 | 44.93 | 0.70 |
| Line-2 | DS | 19.97 | 8.80 | 0.27 | 179.15 | 96.93 | 80.05 | 40.47 | 0.63 |
| Line-3 | DS | 21.04 | 6.24 | 0.24 | 186.64 | 114.52 | 82.24 | 41.04 | 0.68 |
| Line-4 | DS | 27.26 | 5.65 | 0.33 | 164.21 | 109.70 | 78.16 | 40.52 | 0.64 |
| Line-5 | DS | 8.61 | 5.56 | 0.11 | 179.89 | 144.43 | 80.96 | 39.47 | 0.66 |
| Line-6 | DS | 14.64 | 4.36 | 0.26 | 175.14 | 127.59 | 82.59 | 39.69 | 0.65 |
| Line-7 | DS | 19.13 | 4.50 | 0.38 | 162.62 | 112.13 | 82.46 | 41.40 | 0.59 |
| Line-14 | DS | 36.32 | 7.74 | 0.36 | 191.86 | 109.30 | 76.67 | 36.26 | 0.58 |
| Line-17 | DS | 30.33 | 9.96 | 0.29 | 203.12 | 108.09 | - | 28.38 | 0.66 |
| Line-23 | DS | 24.81 | 4.00 | 0.24 | 177.09 | 113.62 | 80.36 | 39.53 | 0.66 |
| Line-26 | DS | 31.52 | 6.29 | 0.23 | 211.54 | 135.85 | 86.07 | 38.87 | 0.71 |
| Line-28 | DS | 29.75 | - | 0.19 | 123.21 | 154.30 | 81.78 | 37.73 | 0.72 |
| Line-30 | DS | 25.62 | 5.89 | 0.44 | 191.16 | 118.50 | 79.46 | 38.89 | 0.66 |
| Line-35 | DS | 33.04 | 7.03 | 0.19 | 200.47 | 136.57 | 82.63 | 34.39 | 0.65 |
| S19-3 | DS | 29.21 | 5.85 | 0.32 | 219.58 | 92.01 | 79.04 | 38.57 | 0.64 |
| DodR | DS | 29.32 | 4.02 | 0.12 | 186.85 | 237.84 | 82.13 | 35.19 | 0.62 |

*Note: A* Photosynthesis rate, *gs* Stomatal conductance, *E* Transpiration rate, *Ci* Intracellular $CO_2$, *WUE* Water use efficiency, *RWC* Relative water content, *CCI* Chlorophyll content index, $F_V/F_M$ Quantum yield of PSII photochemistry, *DS* drought-stressed, - missing value.
