# Supplementary figures and images for: Deciphering the molecular basis for photosynthetic parameters in Bambara groundnut (Vigna subterranea L. Verdc) under drought stress
Source: BMC Plant Biol. 2023 May 30;23:287. doi: 10.1186/s12870-023-04293-w (PMC10228090; doi:10.1186/s12870-023-04293-w)

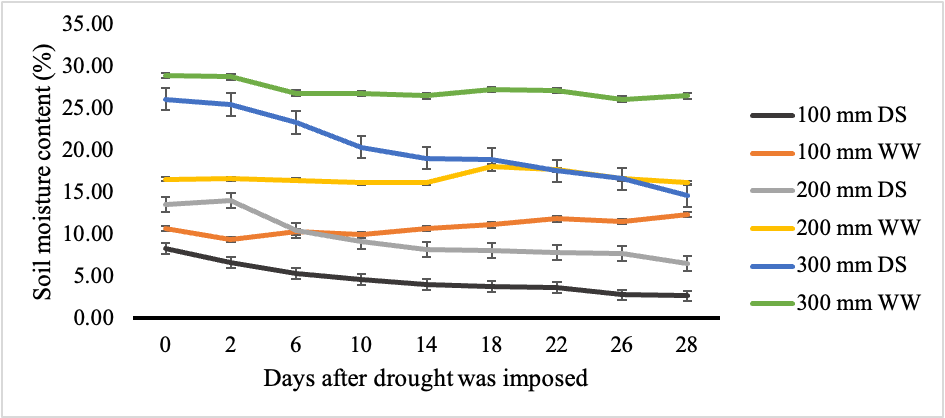

Supplement: Supplementary file 4 — Additional file 4: Supplementary Fig S1. Soil moisture content measurements at depth 100 mm, 200 mm and 300 mm based on PR2 reading (% vol) under drought-stressed (DS) well-watered (WW) conditions. Data represent mean values of soil moisture content during plant growth season in 2019; n = 6. Data represent mean values ± standard error. (Gao et al. 2022 [22]). [file 12870_2023_4293_MOESM4_ESM.png]

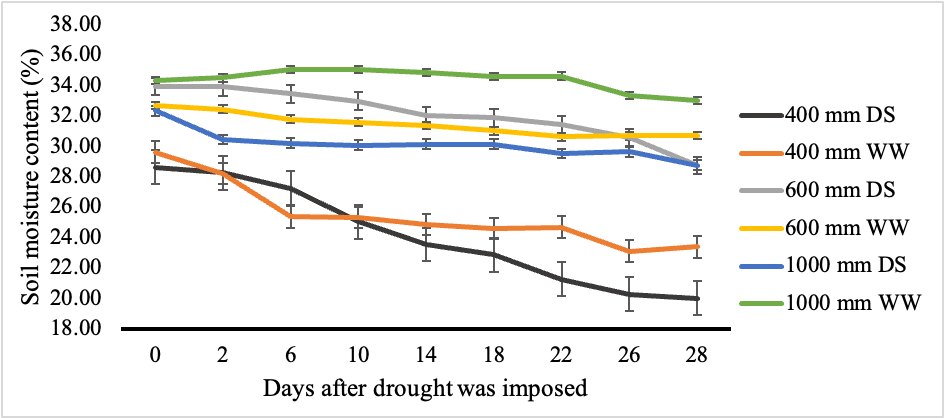

Supplement: Supplementary file 5 — Additional file 5: Supplementary Fig S2. Soil moisture content measurements at depth of 400 mm, 600 mm and 1000 mm based on PR2 reading (% vol) under drought stress (DS) conditions plots and well-watered (WW) conditions plots. Data represent mean values of soil moisture content during plant growth season in 2019; n = 6. Data represent mean values ± standard error (Gao et al. 2022 [22]). [file 12870_2023_4293_MOESM5_ESM.png]
